# Aluminum, Arsenic, Beryllium, Cadmium, Chromium, Cobalt, Copper, Iron, Lead, Mercury, Molybdenum, Nickel, Platinum, Thallium, Titanium, Vanadium, and Zinc: Molecular Aspects in Experimental Liver Injury

**DOI:** 10.3390/ijms232012213

**Published:** 2022-10-13

**Authors:** Rolf Teschke

**Affiliations:** Department of Internal Medicine II, Division of Gastroenterology and Hepatology, Klinikum Hanau, Academic Teaching Hospital of the Medical Faculty, Goethe University Frankfurt, 63450 Hanau, Germany; rolf.teschke@gmx.de; Tel.: +49-6181-21859; Fax: +49-6181-2964211

**Keywords:** aluminum, arsenic, beryllium, cadmium, chromium, cobalt, copper, iron, lead, mercury, molybdenum, nickel, platinum, thallium, titanium, vanadium, zinc, liver injury, ROS, oxidative stress, fenton reaction, pyroptosis, ferroptosis

## Abstract

Experimental liver injury with hepatocelluar necrosis and abnormal liver tests is caused by exposure to heavy metals (HMs) like aluminum, arsenic, beryllium, cadmium, chromium, cobalt, copper, iron, lead, mercury, molybdenum, nickel, platinum, thallium, titanium, vanadium, and zinc. As pollutants, HMs disturb the ecosystem, and as these substances are toxic, they may affect the health of humans and animals. HMs are not biodegradable and may be deposited preferentially in the liver. The use of animal models can help identify molecular and mechanistic steps leading to the injury. HMs commonly initiate hepatocellular overproduction of ROS (reactive oxygen species) due to oxidative stress, resulting in covalent binding of radicals to macromolecular proteins or lipids existing in membranes of subcellular organelles. Liver injury is facilitated by iron via the Fenton reaction, providing ROS, and is triggered if protective antioxidant systems are exhausted. Ferroptosis syn pyroptosis was recently introduced as mechanistic concept in explanations of nickel (Ni) liver injury. NiCl_2_ causes increased iron deposition in the liver, upregulation of cyclooxygenase 2 (COX-2) protein and mRNA expression levels, downregulation of glutathione eroxidase 4 (GPX4), ferritin heavy chain 1 (FTH1), nuclear receptor coactivator 4 (NCOA4) protein, and mRNA expression levels. Nickel may cause hepatic injury through mitochondrial damage and ferroptosis, defined as mechanism of iron-dependent cell death, similar to glutamate-induced excitotoxicity but likely distinct from apoptosis, necrosis, and autophagy. Under discussion were additional mechanistic concepts of hepatocellular uptake and biliary excretion of mercury in exposed animals. For instance, the organic anion transporter 3 (Oat3) and the multidrug resistance-associated protein 2 (Mrp2) were involved in the hepatic handling of mercury. Mercury treatment modified the expression of Mrp2 and Oat3 as assessed by immunoblotting, partially explaining its impaired biliary excretion. Concomitantly, a decrease in Oat3 abundance in the hepatocyte plasma membranes was observed that limits the hepatic uptake of mercury ions. Most importantly and shown for the first time in liver injury caused by HMs, titanium changed the diversity of gut microbiota and modified their metabolic functions, leading to increased generation of lipopolysaccharides (LPS). As endotoxins, LPS may trigger and perpetuate the liver injury at the level of gut-liver. In sum, mechanistic and molecular steps of experimental liver injury due to HM administration are complex, with ROS as the key promotional compound. However, additional concepts such as iron used in the Fenton reaction, ferroptosis, modification of transporter systems, and endotoxins derived from diversity of intestinal bacteria at the gut-liver level merit further consideration.

## 1. Introduction

Heavy metals (HMs), as well as other metallic chemicals, are commonly taken up in small physiological amounts by humans through the food chain or drinking water [1,2,3,4,5,6]. Consequently, they are found at low concentrations in human fluids such as blood, urine, and bile, in addition to selected organs such as the liver. The human body requires HMs as catalysator for a bundle of essential physiological processes, helping to maintain good health condition [1]. Maintaining HM equilibrium in healthy humans is crucial, but well achieved by modifying oral food consumption, intestinal uptake, hepatic storage, biliary excretion, or renal output via the urine. In some genetic diseases, however, HMs contained in normal food accumulates in several organs like the liver, with Wilson disease, for example, causing copper accumulation [7], or hereditary hemochromatosis being known for overload of iron [8]. Primarily healthy humans exposed to high amounts of heavy metals by inhalation were described in industrial workers or the normal population confronted with polluted air [2,3,4,5,6,7]. 

Clinical toxic liver injury in individuals exposed to high amounts of HMs was often insufficiently documented in the literature, as were the underlying pathogenetic steps occurring in the liver. Molecular mechanisms leading to the injury in humans were rarely analyzed, because the liver is embedded in the body and consequently not available for analytical studies due to its behavior as a secret-keeping organ [9], conditions similar to those in human drug-induced liver injury (DILI) [10]. However, liver injury by HMs are well reproducible in animal models, and can be used for studying molecular changes that lead to the injury. Their results may be translated to humans without major limitations, because liver injury by HMs in both, humans and animals, follows a similar mode: it requires high doses of the HMs due to the known dose dependency, and it is predictable in line with intrinsic liver injury definition [11].

This review article focuses on animal models mimicking human liver injury by application of HMs in high doses, elucidating the binding of HMs to structural macroproteins and polyunsaturated fatty acids (PUFA) of hepatic organelles like mitochondria, the generation of mitochondrial and microsomal oxidative stress leading to the production of reactive oxygen species (ROS), and the reduced capacity of antioxidant mechanisms as a defense mechanism against the liver injury. Results from in vitro studies, such as with isolated liver mitochondria or liver parenchymal cell cultures, were also considered. The aim was to update existing mechanistic information of liver injury by HMs, and to connect these data with new developments such as the tentative role of iron, ferroptosis, and gut microbiota that may have consequences for the therapy in humans. 

## 2. Literature Search and Source 

The PubMed database and Google Scholar were searched for articles by using the following key terms: Liver injury or hepatotoxicity and heavy metals and all individual heavy metals listed under the keywords or in the title of this article. These terms were used alone or in combination. The literature search ended on the 30 August 2022. Limited to the English language with a few exemptions, publications from each of the search terms were analyzed in terms of suitability for use in this review article. Publications were complemented from the large private archive of the author. The final compilation consisted of original papers, consensus reports, and review articles, with the most relevant publications included in the reference list of this review.

## 3. Definitions 

### 3.1. Heavy Metals 

HMs and other metals are inorganic chemicals found in the environment, derived from natural sources, or produced by humans in the course of emissions during industrial metallic processes [1,9]. Out of 35 metals, only 23 are defined as HMs due to their high specific density of >5 g/cm^3^ with atomic weight >40.40 [1]. Based on selection criteria regarding quality of published details and availability of valid liver injury data including specifically mechanistic aspects, only the most important HMs were HMs only the most important ones were considered in this article.

### 3.2. Experimental Liver Injury 

Included in this analysis are results of experimental toxicology studies using HMs from animal models, as well as those derived from in vitro experiments. Whereas liver injury is well defined in patients through abnormal liver tests (LTs), with increased serum activities of alanine aminotransferase (ALT) ≥5 times the upper limit of the normal range (ULN) and/or alkaline phosphatase (ALP) ≥2 times of the ULN [9,11], these diagnostic criteria were not applied in animal studies using HMs [9]. Instead, the differences of activities between exposed animals vs. nonexposed controls were commonly reported. Inorganic chemicals, such as most HMs, are problematic because they are not biodegradable via hepatic enzymes and tend towards hepatic bioaccumulation associated with the risk of injury [12]. Excretion of most HMs and their chemical derivatives via urine is marginal and biliary excretion is problematic due to rapid reuptake from the intestine back to the liver via the entero-hepatic circulation. 

## 4. Aluminum 

Aluminum (Al), occasionally also termed as aluminium and commonly classified as a typical prooxidant, caused severe experimental liver injury as aluminum phosphide [13]. It is generally assumed that the sequence of injurious events starts with the overproduction of hepatocellular ROS, generated likely in the course of microsomal and mitochondrial oxidative stress [13,14]. Details of the underlying initial mechanistic steps connected with liver injury by Al were largely unknown. However, based on studies not related to Al, ROS represented a bundle of important intermediate products including toxic radicals such as singlet radical ^1^O_2_, hydrogen peroxide H_2_O_2_, hydroxyl radical HO^•^, alkoxyl radical RO^•^, and peroxyl radical ROO^•^ [9,10,15,16]. Some of these radicals may covalently bind to macromolecular structural proteins or PUFA of liver cell biomembranes such as mitochondria and microsomes. ROS overproduction linked to Al exposure reduced the hepatic glutathione content as well as the activities of glutathione S-transferase, and catalase, with the consequence that the liver is not any more sufficiently protected against toxic ROS [13,14].

Exposure to Al may also result in disruption of the iron homeostasis, with iron overload in the liver [14]. This could enhance ROS production via oxidative stress and increase liver toxicity through the Fenton reaction, known from hereditary hemochromatosis [8,9]. If the focus of liver injury is more on the rough endoplasmic reticulum, a reduction in protein synthesis is to be expected, whereas an injurious focus on the smooth endoplasmic reticulum will impair microsomal drug metabolizing enzymes and reduce the content of cytochrome P450 (CYP) isoforms [13]. Targeting liver mitochondria via ROS due to mitochondrial oxidative stress will cause anaerobic respiration and impair both the tricarboxylic acid cycle function and the electron transport chain reaction, leading to a reduction in ATP production through oxidative phosphorylation [13,14]. Molecular mechanisms of experimental liver injury caused by Al have been exhaustively presented here and may serve as an example for other HMs that trigger experimental liver injury by mechanistic steps broadly similar to those of Al.

## 5. Arsenic 

Arsenic (As) caused experimental liver injury via ROS, whereby specific radicals are generated [3,6,17,18,19,20]. Among these were explicitly the nitric oxide NO^•^, singlet oxygen ^1^O_2_, hydrogen peroxide H_2_O_2_, peroxyl radical ROO^•^, dimethylarsinic radical (CH_3_)_2_As^•^, and dimethylarsinic peroxyl radical (CH_3_)_2_AsOO^•^ [3,6]. The molecular steps leading to these radicals are difficult to firmly establish because the processes are speedy, not allowing quick capturing. The chemical As exists as metalloid (As^0^) [19] and is found in the environment mainly as the inorganic As^3+^, the arsenite [19,21]. Other forms include organic arsenic and arsine (AsH_3_) [19]. 

The liver is the primary target organ not only for toxicity but also for the metabolism of arsenicals with its major metabolic pathway via methylation [19,21], which led to the methylated intermediate of monomethylarsonic acid (MMA) and dimethylarsinic acid (DMA) [21]. Several enzymes localized in the cytosol of the hepatocytes were involved in the metabolism of arsenicals: MMA is synthesized through the arsenite methyltransferase, whereas DMA is synthesized via the DMA methyltransferase [22,23].

Liver injury caused by exposure to As is thought to result from its binding with sulfhydryl groups of enzymes and membrane proteins, conditions that led to cross-linkage [19,21,24,25]. Contributing factors of arsenic liver injury were oxidative DNA, acquired tolerance to apoptosis, enhanced cell proliferation, altered DNA methylation, and genomic instability [21,26]. Subhepatotoxic exposure enhanced preexisting experimental liver injury related to lipopolysachharides (LPS) exposure [27]. Additional metabolomic characterization of liver injury caused by acute As intoxication was described with data derived from the experimental model using zebrafish (*Danio rerio*) [20]. 

Of general interest also for other HMs was the discussion on arsenic toxicology and its data translation from experimental models to human pathology [28]. Proposed was some kind of phenotypic anchoring as a means to unify experimental observations and disease features including clinical outcomes. During a workshop, experimental systems addressing As dose and exposure as well as phenotypic expression relationships and human disease response to prolonged arsenic exposure were studied and identified knowledge gaps. Clinical disease course and outcome is likely dependent on responses of specific cells, individual genetics, and interaction with other toxicants. These conditions may explain some differences between human disease results and experimental data, calling for improved adjusting of experimental study protocols to specific human exposures.

## 6. Beryllium

Beryllium (Be) administrated as Be nitrate caused experimental liver injury [29]. This was associated with a fall in hepatic reduced glutathione content and an increase in lipid peroxidation, interpreted as signs of oxidative stress. Overall, little scientific and clinical interest in Be intoxication and liver injury existed, providing insufficient insight on molecular aspects of Be liver injury [30,31,32,33,34]. Issues of Be were also not discussed in a recent excellent and comprehensive review article focusing on many HMs [9]. 

## 7. Cadmium 

Cadmium (Cd) administration led to experimental liver injury and increased activities of liver antioxidant enzymes like glutathione reductase, glutathione peroxidase [35], and glutathione S-transferase [36]. The associated lipid peroxidation was obviously triggered by iron released from biological membranes [35]. In detail, the toxic Cd^2+^ was bound to the protein metallothionenin (MT) to form the Cd metallothionenin (Cd-MT) complex [37]. This facilitated hepatic cadmium deposition and downgraded glutathione production, accompanied with increased ROS production through oxidative stress and mitochondrial injury. Impaired mitochondrial functions led to metabolic-associated fatty liver disease (MAFLD) due to lipid deposition in the liver. In the further course of events, metabolic-associated steatohepatitis steatosis (MASH) may develop [37].

According to another theory, the acute Cd liver injury may proceed via two pathways [38]. First, the injury is caused by binding of Cd^2+^ to sulfhydryl groups on critical molecules in mitochondria. Thiol group inactivation subsequently caused mitochondrial oxidative stress, mitochondrial permeability transition, and mitochondrial dysfunction. In addition to direct hepatocellular injury based on mitochondrial functional impairment, it was proposed that endothelial cells might be damaged due to hepatic ischemia. Second, the injury might be triggered by Kupffer cell activation with the implication of inflammatory and cytotoxic mediators, such as cytokines and chemokines [38]. Finally, experimental liver oxidative stress induced by Cd was also ascribed to activation of hepatic stellate cells, but more exciting was the observation of Cd uptake and the generation of ROS in hepatocytes detected by mass cytometry and fluorescence microscopy [39]. 

## 8. Chromium 

Chromium (Cr) liver injury strongly depended on its oxidation state with Cr^6+^, syn hexavalent chromium or Cr(VI), and Cr^5+^ as the most toxic species [40]. Inside the hepatocytes, Cr^6+^ is reduced through various reduction pathways to stable Cr^3+^ and to Cr^5+^, which by itself forms a new Cr^5+^ complex, Cr^5+^–BT^2−^, a stable compound that mimicks Cr^6+^ reduction intermediates. Both, Cr^6+^ and Cr^5+^–BT^2−^ led to reversible liver injury. In line with mechanistic steps leading to hepatotoxicity by other heavy metals [6,12,19], the liver injury caused by Cr is primarily due to ROS generated from oxidative stress, which initiates apoptosis of liver cells [41,42,43,44]. Evidence for Cr-promoted oxidative stress in experimental liver injury was provided by an increased malondialdehyde level, protein carbonyl, and advanced oxidation protein product levels [44]. In addition, the antioxidant glutathione, nonprotein thiol, and vitamin C levels were decreased in the liver, associated with reduced activities of the antioxidant enzymes glutathione peroxidase and superoxide dismutase under conditions of increased catalase activities. There is now sufficient experimental evidence that chromium triggers apoptosis and promotes inflammation by inhibiting the deacetylation of SIRT1 [43], which stands for sirtuin (silent mating-type information regulation 2 homolog) as a member of a protein family involved in signaling metabolic regulation. Other, more recent mechanistic proposals focus on signaling processes [45]. For instance, downregulation of Nrf2 signaling may contribute to hepatocyte apoptosis and ROS-dependent liver injury elicited by Cr^6+^, in addition to ASK1/JNK-signaling activity, which was upregulated. 

## 9. Cobalt 

Cobalt (Co) administration caused liver injury to become well studied in animals [46,47,48] by showing initiation of ROS generation via oxidative stress in isolated liver mitochondria that led to permeability transition and apoptosis in hepatocyte cultures [48]. However, as sites of ROS generation in hepatocytes also lysosomes should be considered [6]. Other studies focused on increased lipid peroxidation and alteration of the antioxidant system with a decline in superoxide dismutase and glutathione peroxidase activities or reduced glutathione content in the liver as contributing factors of the liver injury [46]. There was also the note that experimental CoCl_2_ exposure leads to a dose-dependent increase in cyclooxygenase-2 and BAX expressions assessed by immunohistochemistry, findings reported together with a significant decline in hepatic antioxidant enzyme activities and a substantial increase in markers of oxidative stress, but the question of a causal association remains to be answered [49].

## 10. Copper 

Copper (Cu, from the Latin: cuprum) causes in genetically predisposed patients with Wilson disease a special form of liver injurious liver disease due to an excess Cu content in the liver [7], but this has to be differentiated from experimental Cu liver injury [6,50,51,52,53,54,55,56,57]. A broad range of proposals as to how Cu causes mechanistically liver injury have been published, based on human or animal data [50,51]. However, clear pathogenetic concepts on experimental Cu hepatotoxicity from excess consumption are not well characterized [52]. Despite some uncertainties, Cu injury was assumed to be as the result of an interaction between the reduced form of copper and oxygen, leading to ROS comprising superoxide anion, hydrogen peroxide, and hydroxyl radicals, all generated through oxidative stress and seemingly capable of triggering at least partially the injury [6,51]. However, Cu hepatotoxicity is not just oxidative stress, as zinc may be a major cofactor in various cellular processes in this Cu liver injury [50]. Exogenous Cu nanoparticle load also reduced expression of mRNA and several receptors such as aryl hydrocarbon receptor (AHR) and constitutive androstane receptor (CAR); both affect mainly the expression of CYP 1A isoform, and pregnane X receptor (PXR), which express the CYP 2C and 3A isoforms [57]. Modification of CYP isoforms is closely related to changes in key transcription and protein kinases. There is also the notion that free, unbound Cu is the most toxic form of this metal [52], and Cu liver injury proceeds via the Fenton reaction, which uses iron and H_2_O_2_ and results in toxic radical formation [6]. It remains to be established whether animal models [53,54], including the goldfish model [55] or the zebrafish model [56], can contribute to close the existing mechanistic gaps in excess Cu liver injury. There are several animal models like special dog or Long Evans strains with congenital disturbances of metabolic Cu handling, suitable for studies regarding human Wilson disease but not for experimental liver injury due to overdosed Cu via exogenous administration. 

## 11. Iron 

Iron (Fe, from Latin: ferrum) caused experimental liver injury in animals [6,58,59,60,61,62]. This condition has to be differentiated from hereditary hemochromatosis, a human disease based on a genetic abnormality causing increased Fe levels preferentially in the liver [8,62]. Mechanistic steps leading to experimental liver injury by Fe administrated in excess suggested a key role of hepatocellular oxidative stress generating ROS as responsible intermediate for apoptosis, lipid peroxidation, and reduced superoxide dismutase activity [58,59]. Studies on iron metabolism using the zebrafish model failed to contribute new aspects to the mechanistic steps [56]. In addition, proteomic analysis in mice with hepatic iron overload suggested dysregulation of the urea cycle, impairment of fatty acid oxidation, changes in the methylation cycle [60], and immune cell activation [58]. Free iron is extremely toxic to cells, but several protective mechanisms exist in the cells aiming to bind the iron, including the transferrin in the blood [61]. In more detail, the toxicity of iron in animal studies is related to the ability of ferrous iron to interact with H_2_O_2_, allowing the generation of the highly reactive hydroxyl radical through a specific mechanism, the Fenton reaction [62]. However, whether the Fenton reaction is operative in humans remains to be established. 

## 12. Lead 

Lead (Pb for Latin plumbum) overdose in animals caused experimental liver injury. Elucidating its pathogenic background was challenging as some issues remain controversial, although many studies have been published [63,64,65,66,67,68,69,70,71,72,73]. Consensus exists among experts that ROS as a product of oxidative stress plays a major role in initiating the injury process [12,63,64,65,66,67,68,69]. Pb binds to sulfhydryl groups of structural proteins or any cytosolic protein such as glutathione, thereby reducing the antioxidant defense property and enhancing the Pb toxicity [64,65,67,68,70,71] through lipid peroxidation of cell membranes, such as of mitochondria or endoplasmic reticulum [12,64,65,66,70,72]. The high affinity of Pb to protein sulfhydryl groups resulted in lower activities of a number of enzymes like catalase, glutathione peroxidase, glucose-6-phosphate dehydrogenase, and superoxide dismutase [65,72]. Contrary results of increased enzyme activities have been reported, though the reason for this discrepancy remains unexplained [71,73]. Clarifying studies on Pb liver injury using the zebrafish model have not yet been done [56].

## 13. Mercury 

Mercury, or Hg for formerly named hydrargyrum from the Greek words hydor, water, and argyros syn silver [12], caused experimental liver injury through mechanistic steps that start with the hepatocellular generation of ROS, resulting in disruptions of the antioxidant defense system and lipid peroxidation formation [12,74]. Other pathogenetic aspects were provided by results obtained from *Bryconamzonicus*, a freshwater fish [12] or Zebrafish gills after exposure to HgCl_2_ [75]. Additional mechanistic studies focused on hepatocellular uptake and biliary excretion of Hg in exposed animals [76]. The organic anion transporter 3 (Oat3) and the multidrug resistance-associated protein 2 (Mrp2) were involved in the hepatic excretion of toxins and drugs as well as in the hepatic handling of mercury. Oat3 and Mrp2 expression was assessed by immunoblotting. Hg treatment decreased the expression of Mrp2 in both sexes and reduced the expression of Oat3 only in males, suggesting that Hg impairs its own biliary excretion. At the same time, Hg administration caused a decrease in Oat3 abundance in the hepatocyte plasma membranes in male animals that would limit the uptake of Hg ions into the liver, protecting them partially from Hg accumulation in the liver and reducing the liver injury. Female animals were more susceptible to Hg liver injury compared with male ones, findings most likely attributable to differences in biliary Hg excretion and its hepatocellular uptake.

## 14. Molybdenum 

Molybdenum (Mo) exposure resulted in experimental liver injury, as evidenced by hepatocyte apoptosis with involvement of a mitochondrial pathway [77]. Mo downregulated levels of superoxide dismutase and catalase in the hepatocytes, associated with upregulation of malondialdehyde, nitric oxide, and nitric oxide synthase. Overall data on experimental liver injury induced by Mo were limited [6,77,78]. Finally, Mo can replace intracellular Cu and led to localized Cu deficiency [6], which may contribute to liver injury observed following ingestion of Mo in excess.

## 15. Nickel 

Nickel (Ni) overdose led to liver injury in animals, its pathogenetic concept goes primarily back to ROS generated from oxidative stress, leading to lipid peroxidation [12,79,80,81]. This was associated with both, depletion of the hepatic glutathione levels [12] and Ni accumulation in the liver [82]. Liver injury by Ni nanoparticles was also triggered by nitrative stress, causing apoptosis and inflammation [83]. Evidence for the nitrative stress concept was provided by augmented specific stress markers like iNOS (inducible nitric oxide synthase) or NO (nitric oxide). According to other proposals, Ni may also initiate inflammatory and apoptosis processes through activation of transcription factors [6]. Ferroptosis syn pyroptosis was introduced as a mechanistic concept in Ni liver injury [84]. NiCl_2_ caused increased iron content in the liver, upregulation of cyclooxygenase 2 (COX-2) protein and mRNA expression levels, downregulation of glutathione eroxidase 4 (GPX4), ferritin heavy chain 1 (FTH1), nuclear receptor coactivator 4 (NCOA4) protein and mRNA expression levels. The conclusion was reached that Ni may cause hepatic injury through mitochondrial damage and ferroptosis [84]. Ferroptosis is defined as iron-dependent cell death, similar to glutamate-induced excitotoxicity but distinct from apoptosis, necrosis, and autophagy, and is triggered by inhibition of cystine uptake, whereby reduced cystine uptake leads to the production of lethal lipid ROS [85]. Similarly, pathogenetic involvement of ferroptosis was discussed in a variety of diseases [85,86,87,88,89] such as high-fat diet-induced hepatic lipotoxicity [87], non-alcoholic fatty liver disease (NAFLD) [88,89], DILI [89], and in liver injury of patients with COVID-19 infections [90], partly attributed to DILI with verified diagnosis using RUCAM (Roussel Uclaf Causality Assessment Method) [91]. Overall, the ferroptosis concept is highly complex and seems different from the Fenton reaction regarding the sequence of events, although iron is involved in both. First, the Fenton reaction with presence of iron allows the formation of radicals. Second, the radicals combine with PUFA to form lipid peroxides through pathways involving iron containing enzymes, a process now called ferroptosis.

## 16. Platinum 

Platinum (Pt) in the form of the clinically-used anticancer drug oxaliplatin (OXA) caused experimental liver injury [92,93] with focal liver cell necrosis, but without the typical signs of hepatic sinusoidal obstruction syndrome (HSOS) [92] known from patients with cancer disease treated with OXA [94] or consumers of plants of herbal medicines containing 1,2-unsaturated pyrrolizidine alkaloids (PAs) [95]. The results from the animal model of liver injury caused by OXA suggested that oxidative stress has an important role in its pathogenesis [92].

## 17. Thallium 

Thallium (Tl) administration results in experimental liver injury because the liver is the preferred site of its storage, and hepatic mitochondria are the most important target subcellular organelles within the hepatocytes for the Tl injury [96], as evidenced by impaired mitochondrial fatty acid metabolism [97]. Isolated mitochondria treated with Tl in vitro exhibited marked elevation in oxidative stress parameters, accompanied by increased mitochondrial ROS generation, adenosine triphosphate (ATP) depletion, impaired oxidation of reduced glutathione (GSH), and mitochondrial membrane potential (MMP) collapse [96]. Together with mitochondrial outer membrane rupture and swelling, and disruption of the mitochondrial respiratory chain, these events trigger hepatocellular death signaling via opening of mitochondrial permeability transition pore. 

## 18. Titanium 

Titanium (Ti) administration in animals led to experimental liver injury, whereby mechanistic steps causing the injury were confined to a reduction in antioxidative properties related to superoxide dismutase, catalase, and glutathione [98]. A theoretical involvement of ROS was also discussed [98,99]. Additional liver metabolomics analysis showed that 29 metabolites and two metabolic pathways changed significantly [99]. Most importantly and shown for the first time for experimental liver injury caused by HMs, Ti changed the diversity of gut microbiota and modified their metabolic functions, leading to increased generation of LPS, which as endotoxins may trigger and perpetuate the liver injury [99] in a similar way shown as gut-liver axis for clinical ALD in humans [100,101]. 

## 19. Vanadium

Vanadium (V) intoxication resulted in experimental liver injury, interpreted in line with oxidative damage as evidenced by the modification of usual parameters such as hepatic superoxide dismutase and glutathione peroxidase activities [102]. The hepatocelluar oxidative injury proceeded via a Fenton-like reaction with generation of free radicals [6]. 

## 20. Zinc 

Zinc (Zn) toxicity affected animals, providing experimental liver injury [103,104,105] with pathogenetic details focusing on zinc oxide nanoparticles causing oxidative stress indicated by an increased lipid peroxidation, leading to DNA damage and apoptosis of hepatocytes [103]. Oxidative stress with augmented ROS generation was associated with reduced availability of protective antioxidant defense mechanisms [104]. Hepatic Zn toxicity was also characterized by alteration of mitochondrial metabolism and decreased ATP production in hepatocytes [105]. Interesting details of the intracellular Zn handling through genetically determined Zn transporters have been published using the zebrafish model [56].

## 21. Varia 

The current literature search revealed little data on evidence or mechanisms of experimental liver injury by HMs like gold (aurum) [2,6,12,106], manganese [2,6,12,107], selenium [2,6,12,108], silver [2,6,12,109], tin and uranium [2,6,12]. As a consequence of data rarity, these HMs were not considered in his review.

## 22. Conclusions

Animals exposed to high amounts of HMs or their chemical derivatives are confronted with the issue of getting rid of these potentially toxic chemicals, which are only partially excreted via the urine or bile, with biliary HMs being reabsorbable from the intestine and transferred back to the liver via the entero-hepatic circulation. The accumulation of HMs in the liver facilitates direct contact with membranes of subcellular organelles like mitochondria and the endoplasmic reticulum of their structural macroproteins and PUFA. As a result of this interaction, ROS is formed with various toxic radicals, producing lipid peroxides from PUFA peroxidation and initiating or perpetuating liver injury because of less-functioning antioxidant systems like reduced glutathione, catalase, or superoxide dismutase. Iron is involved via the Fenton reaction as well as via pathways with iron-containing enzymes, now called ferroptosis. Endotoxins derived from intestinal bacteria may play a contributory role in the injury. In essence, initial mechanistic steps of experimental liver can partially be traced back to the Fenton reaction promoting the generation of ROS and lipid peroxidation of membrane PUFA, to pathways involved in ferroptosis, and intestinal bacterial endotoxins. Future research should clarify the mechanistic events leading to hepatic ROS production using these excellent HM animal models. Promising are also studies on the iron liberation from existing iron-containing enzymes or other yet unknown sources.
